# Telehealth-supervised exercise in systemic lupus erythematosus: A pilot study

**DOI:** 10.1177/09612033231157073

**Published:** 2023-02-20

**Authors:** Stephanie Frade, Sean O’Neill, Samantha Walsh, Chloe Campbell, David Greene, Stephen P. Bird, Melainie Cameron

**Affiliations:** 1 School of Health and Medical Sciences, Faculty of Health and Engineering and Sciences, 95789 University of Southern Queensland, Ipswich, Australia; 2 Department of Rheumatology 247197 Institute of Bone and Joint Research, Kolling Institute, University of Sydney, Sydney, Australia; 3 School of Behavioural and Health Sciences 95582 Australian Catholic University, Strathfield, Australia

**Keywords:** systemic lupus erythematosus, exercise, autoimmune disease, telehealth, COVID-19

## Abstract

**Objectives:**

To explore the feasibility and effectiveness of telehealth-supervised exercise for adults with Systemic lupus erythematosus (SLE).

**Methods:**

This was a non-randomised controlled pilot trial comparing telehealth-supervised exercise (8 weeks, 2 days/week, 45 min, moderate intensity) plus usual care with usual care alone. Mixed methods were used to assess change in fatigue (FACIT-fatigue), quality of life (SF36), resting fatigue and pain (11-point scale), lower body strength (five-time sit-to-stand) and endurance (30 s sit-to-stand), upper body endurance (30 s arm curl), aerobic capacity (2 min step test), and experience (survey and interviews). Group comparison was performed statistically using a two-sample T-test or Mann–Whitney U-test. Where known, we used MCID or MCII, or assumed a change of 10%, to determine clinically meaningful change within groups over time. Interviews were analysed using reflexive thematic analysis.

**Results:**

Fifteen female adults with SLE were included (control group *n* = 7, exercise group *n* = 8). Statistically significant differences between groups, in favour of the exercise intervention, were noted for SF36 domain emotional well-being (*p* = 0.048) and resting fatigue (*p* = 0.012). There were clinically meaningful improvements over time for FACIT-fatigue (+6.3 ± 8.3, MCID >5.9), SF36 domains physical role functioning (+30%), emotional role functioning (+55%), energy/fatigue (+26%), emotional well-being (+19%), social functioning (+30%), resting pain (−32%), and upper body endurance (+23%) within the exercise group. Exercise attendance was high (98%, 110/112 sessions); participants *strongly agreed* (*n* = 5/7, 71%) or *agreed* (*n* = 2/7, 29%) they would do telehealth-supervised exercise again and were satisfied with the experience. Four themes emerged: (1) ease and efficiency of exercising from home, (2) value of live exercise instruction, (3) challenges of exercising at home, and (4) continuation of telehealth-supervised exercise sessions.

**Conclusion:**

Key findings from this mixed-method investigation suggest that telehealth-supervised exercise was feasible for, and well-accepted by, adults with SLE and resulted in some modest health improvements. We recommend a follow-up RCT with more SLE participants.

## Introduction

Systemic lupus erythematosus (SLE) is a chronic, multisystem, autoimmune disease characterised by an immune response to self-antigens.^1,2^ Common manifestations of SLE include fatigue, affecting up to 80% of patients,^3^ arthritis, myalgia, serositis, and nephritis.^1^ People with SLE are less physically active than people without SLE.^4^ Sixty percent of people with SLE do not meet World Health Organisation (WHO) recommendations for physical activity (PA).^4^ Additionally, physical inactivity increases the risk of developing common comorbidities such as osteoporosis^5^ and atherosclerotic cardiovascular disease (CVD).^6,7^ Regular, moderate intensity exercise is demonstrated to be a safe and effective adjunctive therapy to improve aerobic capacity, fatigue, depression, and physical function in people with SLE.^2,8,9^ Jeyasingham and colleagues surveyed 55 adults with SLE^10^ and found that most (*n* = 49, 89%) reported some barriers to engagement in regular exercise; reasons included fatigue (*n* = 39, 71%), lack of time (*n* = 27, 49%), weather conditions (*n* = 18, 33%), and lack of motivation (*n* = 17, 31%). Promisingly, most participants (*n* = 48, 87%) were willing to change their daily routine to include more exercise.^10^ Additionally, Dickson and colleagues^11^ surveyed 1113 adults with rheumatologic diseases to assess perceived influences of the COVID-19 pandemic on PA, revealing additional barriers. Over half of participants (55.5%) reported engaging in less PA, followed by unchanged PA (26.6%), and increased PA (15.3%) since the start of the pandemic; reasons included increased overall fear/anxiety (33.5%), lack of motivation (32.4%), and contracting coronavirus infection (32.1%). Most participants reported that they did not meet their exercise goals during the 2020/2021 years of COVID-19 pandemic (67.2%).^11^

Telehealth is a possible strategy for delivering exercise interventions for people with SLE that may alleviate some of the reported barriers (i.e. exercise performed in the comfort of the participants’ home, requiring no additional travel time and energy, and supervised to ensure safety, and increase motivation). Telehealth exercise interventions targeting fitness have proved effective and safe in other populations, including cardiopulmonary diseases^12^ and multiple sclerosis.^13^ Galloway and colleagues^14^ trialled telehealth using real-time video as an exercise delivery mode for twenty-one people recovering from stroke and found that feasibility and satisfaction were high; 95% of participants rated usability favourably, and 95% ‘enjoyed’ telehealth exercise sessions and ‘would recommend them to others’.^14^ Telehealth-supervised exercise does not appear to have been trialled for people with SLE. Therefore, in this pilot study we aimed to explore the feasibility and effectiveness of individually supervised telehealth exercise for adults with SLE adjunctive to their usual care.

## Methods

### Study design

This study was a non-randomised controlled pilot trial conducted between September 2021 and December 2022. This study was approved by the University of Southern Queensland (USQ) Human Research Ethics Committee (ethics application number: H21REA052) and registered with Australia and New Zealand Clinical trial registry (ACTRN12622000063718).

### Participants

Participants were recruited through advertisement within a tertiary hospital rheumatology department and the Lupus New South Wales (NSW) association. Following initial screening, those who met the inclusion criteria and signed consent were included in the study. Inclusion criteria were age ≥ 18 years, diagnosis of SLE according to the European League Against Rheumatism (EULAR)^15^ or American College of Rheumatology (ACR) criteria for SLE,^16^ and deemed safe to exercise by principal investigator (SF) who is an accredited exercise physiologist (AEP). Exclusion criteria were those who were pregnant, or had active lupus nephritis, myocarditis, or pericarditis, or otherwise deemed unsafe to exercise.

### Interventions

Participants in the exercise group underwent an 8 week, 2 days per week, 45 min, individually supervised telehealth exercise program. All sessions were conducted in real-time on Zoom (Zoom Video Communications, Inc, CA, USA) by an AEP (i.e. SF delivered one session per week, and a trained research assistant delivered one session per week). Exercise was performed at moderate intensity, with a rating of perceived exertion (RPE) between 3 and 4 out of 10, in accordance with the American College of Sports Medicine (ACSM) intensity guidelines,^16^ which was monitored through-out the program. All participants were allocated 48 hours of relative rest (i.e. no structured exercise) between the two sessions. The session comprised of a 10 -min seated mobility warm up, 30-min strength circuit, and a 5-min static stretching and breathing cool down. The circuit was designed in accordance with the ACSM resistance training guidelines^16^ for muscular strength, with exercise volume comprising 2–4 sets and 8–12 repetitions, 1 min rest between each set, and inclusion of 6–8 exercises focusing on major muscle groups. The program was structured as a circuit, with 6–8 exercises comprising 1 set, incorporating fundamental movements: push, pull, squat, lunge, locomotion, and rotation. Resistance included body weight, available items in participants’ home, and two resistance bands which were sent to participants. Exercise volume progressed over the 8 weeks consistently between participants (i.e. 2 sets progressed to 3 sets, 8 repetitions progressed to 12 reps); however, RPE was used as the primary tool to substantiate an increase or decrease in intensity (i.e. increasing tension on the resistance bands) to ensure participants maintained the desired RPE. All participants maintained their usual care during the duration of the intervention. Participants in the control group continued with their usual care; we did not ask control participants to stop their usual exercise routines, nor did we prescribe any new exercises.

### Outcomes

Baseline and post-intervention testing were conducted by a blinded investigator (SW), also an AEP. Self-reported questionnaires were sent to the participants to complete, and exercise tests were conducted in real-time on Zoom. Data were stored electronically on a university password-secured OneDrive folder.

#### Pain and fatigue

An 11-point scale (e.g. 0 = no pain to 10 = maximum pain) was used to measure participants’ self-reported resting pain and fatigue. Lower scores indicate less pain and fatigue (lower scores are better). This scale has been visually adapted from the 10-point Borg RPE scale, with good reliability (0.898) and correlation to the visual analogue scale (rs = 0.754, *p* < 0.01).^17^ Each number on the scale included a description (e.g. 1 = just noticeable, ‘my pain is hardly noticeable’). This scale was also used to monitor the exercise program. A change of 15% (mean change/baseline × 100) has been identified as the minimally clinically importance difference (MCID) for pain in people with chronic musculoskeletal pain.^18^

#### Fatigue

The Functional Assessment of Chronic Illness Therapy Fatigue Scale (FACIT-F) was used to measure self-reported fatigue. Functional Assessment of Chronic Illness Therapy Fatigue Scale is reliable (α > 0.95) and has been validated in SLE (ρ 0.81).^19^ Functional Assessment of Chronic Illness Therapy Fatigue Scale-F (version 4) is a 13-item questionnaire that uses a 5-point Likert-type response scale (0 = not at all; 1 = a little bit; 2 = somewhat; 3 = quite a bit; and 4 = very much), with scores ranging from 0 to 52 (higher scores indicating less fatigue). Goligher et al.^20^ derived 5.9 points as the MCID for the FACIT-F scale in SLE.^20^

### Quality of life

The RAND 36-Item Health Survey (version 1.0)^21^ (SF36) was used to measure self-reported quality of life (QOL) on eight health domains including physical and emotional limitations, fatigue/energy, emotional well-being, social functioning, pain, and general health. SF36 has good reliability as a measure of QOL^21^ and has been used to measure QOL in various exercise intervention studies in SLE.^22–27^ Scores for each domain range from 0 to 100, with a higher score defining a more favourable health state.^28^ An MCID has not been identified for each individual domain.

#### Lower body endurance

A 30-second sit-to-stand (30sSTS) test was used to measure lower body muscular endurance because of its reliability in telehealth (ICC 0.989),^29^ excellent test–retest reliability in community-dwelling older adults (men, ICC 0.84 and women, ICC 0.92),^30^ and validity correlating to weight-adjusted leg press performance (men, *r* = 0.78 and women, *r* = 0.71).^30^ Lower limb muscular endurance is the ability of the lower limb muscles to perform repetitive contractions against a force for an extended period of time.^16^ This test involves the participant standing up and sitting down as many times as possible in 30-seconds, whereby the greater number of repetitions completed indicates greater lower limb muscular endurance (higher scores are better). The minimal clinically important improvement (MCII) for a 30sSTS is 2.6.^31^

#### Lower body strength

A five-time STS (5TSTS) was used to measure lower body muscular strength because of its reliability in telehealth (ICC 0.990),^29^ excellent test–retest reliability in older adults with hip or knee osteoarthritis (ICC 0.96),^32^ and good validity when compared to the timed up and go (TUG) test in older adults (*r* = 0.64).^33^ Lower limb strength is the ability of the lower limb muscles to exert a maximum force against an object external to the body, or own body weight, in one maximum effort of the lower body muscles.^16^ This test assesses the time it takes to stand up and sit down five times, whereby the less time it takes to complete five repetitions, the greater the lower limb strength (lower scores are better). The MCID for a 5TSTS is 2.3 s.^34^

#### Upper body endurance

A 30-second arm curl test (30sAC) was used to measure upper body muscular endurance because of its reliability in telehealth (ICC 0.992),^29^ good test–retest reliability (ICC 0.80–0.81) in an older population,^35^ and good validity (*r* = 0.84 for men and *r* = 0.79 for women) when compared to composite strength measures (1-repetition max biceps, chest, and upper back).^36^ Upper body endurance is the ability for upper body muscles to continue contracting against external resistance for an extended period.^37^ To perform this test successfully via telehealth, participants were instructed to do as many arm curls (bending the arms simultaneously towards the body at the elbow) as they could using available household items (e.g. dumbbell, water jug, and cans) in 30-seconds. The greater number of repetitions completed indicates greater upper limb endurance (higher scores are better). An MCID has not been identified for this test.

#### Aerobic capacity

The 2 minute step test (2MST) was used to measure aerobic capacity because of its reliability in telehealth (ICC 0.999),^29^ excellent test–retest reliability (ICC = 0.95),^38^ and validity correlating to the 6-min walking test (*p* = 0.04).^39^ Aerobic capacity is the measure of the body’s ability to use oxygen from the atmosphere and produce energy for muscle cells.^16^ For this test, participants were instructed to stand perpendicular to the wall and march in one place as many times as they could in 2 min, whereby the higher number of repetitions indicates greater aerobic capacity (higher scores are better). The number of knee lifts performed on the right leg in 2 min was recorded. An MCID has not been determined for this test.

#### Participant feedback

**Figure 1.**
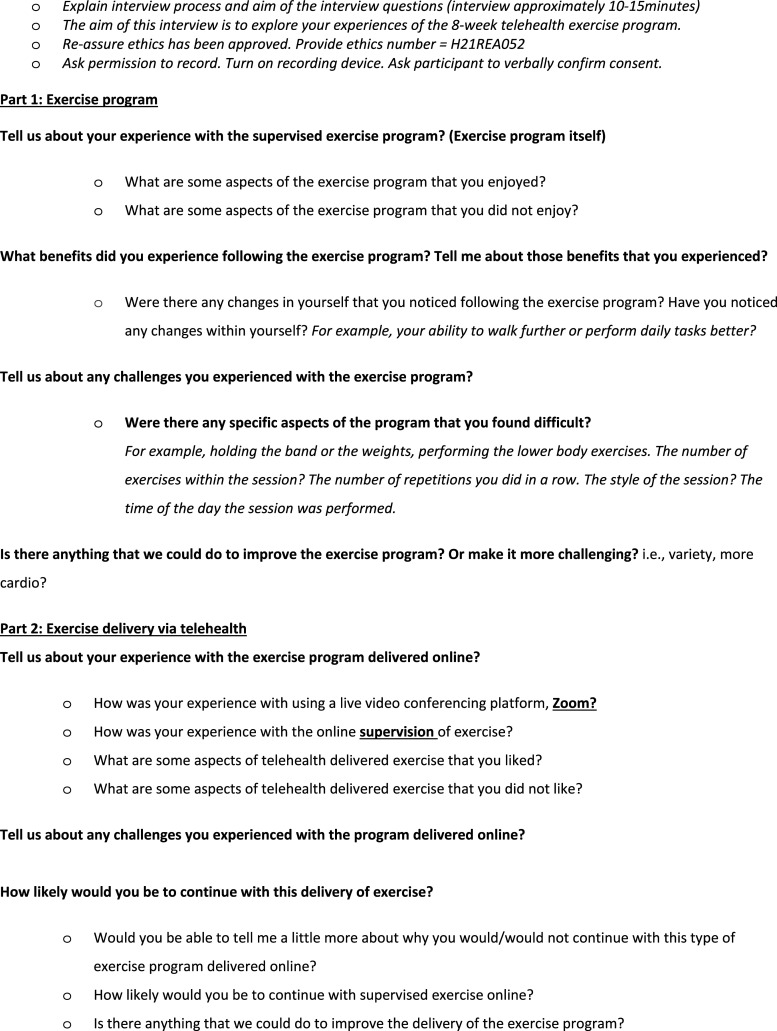
Post-intervention interview framework.

Participants who completed the exercise program provided quantitative feedback about the telehealth-supervised exercise program via a face-validated questionnaire used by Galloway and colleagues in stroke,^14^ with minor modifications made to better reflect our study design and participants. The questionnaire was sent electronically to participants post-intervention, and data were generated using Qualtrics XM® software (Provo, UT, USA), presented as the number and percentage of respondents. Participants also provided qualitative feedback during a 15-minute semi-structured interview (Figure 1), conducted and audio-recorded on Zoom, transcribed using Otter.ai transcription software (Mountain View, CA), and analysed using NVivo software (QSR International Pty Ltd, VIC, AUS). A six-phase reflexive thematic methodology was used to analyse themes.^40^ Key quotations from the transcripts were selected to illustrate themes and de-identified by an alphanumeric code that represents their disease duration (i.e. F12), consistent with the reporting of quantitative data.

### Attendance

Attendance to the exercise intervention was calculated by taking the number of attended sessions as a percentage of the total number of scheduled sessions.^41^

### Statistical analysis

The sample size was calculated using SF36 fatigue/energy domain results from Tench (2003)^27^ who explored the effect of exercise on fatigue; considering an effect size (Cohen’s d) of 2.18, alpha level of 0.05, and a power of 90%, a minimum of 6 participants per group (total of 12 participants) was required. Missing data were imputed using the last measure carried forward method. Descriptive (mean, standard deviation, median, and interquartile ranges) and statistical analyses were performed using Microsoft Excel (Microsoft Corporation, Redmond, WA). Shapiro–Wilks test was used to examine distribution of data. End of intervention measures are reported as change scores from baseline. Comparisons between groups were performed using a two-sample t-test for normally distributed data and a Mann–Whitney U test for non-normally distributed data. Alpha (α) level of 0.05 was pre-determined as the arbiter of statistical significance (*p* ≤ 0.05) for inferential tests. Where known, we used MCID or MCII, or assumed a change of 10% (mean change/baseline ×100),^42^ to determine clinically meaningful change within groups.

## Results

### Participant characteristics

Fifteen adults with SLE expressed interest in the study and were all eligible (control group *n* = 7, exercise group *n* = 8); one participant in the exercise group withdrew due to other health complications. All control group participants engaged in regular exercise (e.g. walking, resistance, and yoga), *n* = 4/7 had fibromyalgia overlap, medications included *n* = 5/7 hydroxychloroquine, *n* = 3/7 immunosuppressants, and *n* = 1/7 corticosteroids. Most (*n* = 5/7) exercise group participants engaged in regular exercise (e.g. walking, running, and stationary cycling), *n* = 4/7 had fibromyalgia overlap, medications included *n* = 6/8 hydroxychloroquine, *n* = 4/8 immunosuppressants, and *n* = 3/8 corticosteroids. There were no reported changes to their prescribed medication upon completion of the exercise program. No participants were on biologic therapies. All participants had joint (*n* = 15/15), skin (*n* = 11/15), and/or renal involvement (*n* = 8/15). The four most common symptoms reported were fatigue (*n* = 14/15), joint pain (*n* = 12/15), muscle aches (*n* = 10/15), and brain fog (*n* = 12/15) (Table 1).

**Table 1.** Demographic characteristics and baseline data for the two groups.

Variable	Control group (*n* = 7)				Exercise group (*n* = 8)				*p*-Value
	Mean	SD	Median	IQR	Mean	SD	Median	IQR	
Age (years)	41	11	38	8	48	18	48	30	0.352
Disease duration (years)	7	6	7	9	12	8	11	11	0.222
RHR (beats/min)	65	5	66	8	70	11	68	18	0.312
Rpain (0–10 scale)	3	1	3	2	2	1	2	2	0.304
Rfatigue (0–10 scale)	3	2	3	3	3	2	3	3	0.441
FACIT-F (0–52 scale)	22	9	24	13	26	15	24	24	0.518
FTSTS (seconds)	12	2	13	4	13	4	13	5	0.656
30sSTS (repetitions)	13	3	12	4	13	3	13	4	0.873
30sAC (repetitions)	16	4	15	5	16	5	16	6	0.916
2MST (repetitions)	62	19	65	32	70	17	73	24	0.43

RHR: resting heart rate; Rpain: resting pain; Rfatigue: resting fatigue; FACIT-F: functional assessment of chronic illness therapy (fatigue measurement system); 5TSTS: five-time sit to stand test; 30sSTS: 30 s sit to stand test; 30sAC: 30 s bicep/arm curl test; 2MST: 2 min step test.

### Quantitative results

#### Pain and fatigue (11-point scale)

There was no statistically significant difference between the exercise and control group for resting pain (*p* = 0.633). There was a statistically significant difference in resting fatigue (*p* = 0.012) between the exercise (mean change −0.8 ± 1.5) and control group (mean change +1.4, ±1.4), favouring the exercise intervention. There was a clinically meaningful improvement in resting pain (−32%) over time within the exercise group (mean change −0.6 ± 0.7). There was no clinically meaningful improvement in resting pain over time for the control group and resting fatigue over time within both groups (Table 2).

**Table 2.** Comparison of exercise versus control group changes from baseline.

Variable	Control group (*n* = 7)				Exercise group (*n* = 8)				*p*-Value
	Mean	SD	Median	IQR	Mean	SD	Median	IQR	
RHR (beats/min)	6.9	11.7	4	15	−3.8	7.8	−4	7.3	**0.057**
Rpain (0–10 scale)	−0.3	1.8	−1	3	−0.6^a^	0.7	−0.5	1	0.633
Rfatigue (0–10 scale)	1.4	1.4	1	2.5	−0.8	1.5	−0.5	3	0.012^b^
FACIT-F (0–52 scale)	−0.6	7.9	−1	16	6.3^c^	8.3	4	12.3	0.128
5TSTS (seconds)	−1.5	1.8	−2	3	−1	1.7	−1	2.7	0.574
30sSTS (repetitions)	1	2.8	1	5	1.1	3.2	1	2.5	0.937
30sAC (repetitions)	1.7	1.5	2	3	3.8	2.9	3.5	4.5	0.121
2MST (repetitions)	5	8.8	8	15	3	12.5	−2.5	23.3	0.745

RHR: resting heart rate; Rpain: resting pain; Rfatigue: resting fatigue; FACIT-F: functional assessment of chronic illness therapy (fatigue measurement system); 5TSTS: five-time sit to stand test; 30sSTS: 30 second sit to stand test; 30sAC: 30 second bicep/arm curl test; 2MST: 2 minute step test.

^a^Clinically meaningful improvement over time within group (>15% change).

^b^Statistically significant difference between groups (*p* < 0.05).

^c^Clinically meaningful improvement over time within group (MCID >5.9 points).

#### Fatigue (FACIT-F)

There was no statistically significant difference between the exercise and control group (*p* = 0.128). There was a clinically meaningful improvement in fatigue over time within the exercise group (mean change +6.3 ± 8.5). However, the median change (+4 ± 12.3) did not meet this MCID. There was no clinically meaningful improvement over time within the control group (Table 2).

#### Quality of life (SF36)

There was a statistically significant difference between groups, in favour of the exercise intervention, for the SF36 domain emotional well-being (*p* = 0.048) only. There were clinically meaningful improvements in physical role functioning (+30%), emotional role functioning (+55%), energy/fatigue (+26%), emotional well-being (+19%), and social functioning (+30%), over time within the exercise group. There were clinically meaningful improvements in physical role functioning (167%) and energy/fatigue (11.6%) over time within the control group (Table 3).

**Table 3.** SF36 domain score for the two groups before and after 8 weeks of intervention.

SF36 domain	Control group (*n* = 7)								Exercise group (*n* = 8)								*p*-Value
	Baseline		Post		Change				Baseline		Post		Change				
	Mean	SD	Mean	SD	Mean	SD	Median	IQR	Mean	SD	Mean	SD	Mean	SD	Median	IQR	
Physical functioning	65.7	33.2	66.4	30.6	0.7	44.8	5	10	68.1	30.9	67.5	34.7	−0.6	6.8	0	8.75	0.105*
Physical role functioning	10.7	19.7	28.6	41.9	17.9^a^	53.5	0	100	31.3	43.8	40.6	44.2	9.4^a^	29.7	0	18.75	0.862*
Emotional role functioning	61.9	48.8	52.4	50.4	−9.5	49.9	0	33.34	37.6	45.2	58.3	42.7	20.8^a^	35.4	16.5	58.34	0.193
Energy/fatigue	25	12.9	27.9	18.9	2.9^a^	19.8	10	40	36.3	25.6	45.6	25.7	9.4^a^	17.2	10	22.5	0.506
Emotional well-being	60.6	18.7	59.4	20.6	−5.7	14.9	−8	16	57	15.8	68	14.8	11^a^	14.6	10	27	0.048^b^
Social functioning	39.3	32.6	41.1	35.9	1.8	26.4	0	50	42.2	24.9	54.7	32	12.5^a^	23.1	6.25	21.88	0.418
Pain	51.8	33	51.1	18.3	−0.7	23.8	−10	35	64.4	24.4	60.9	22	−3.4	18.4	−6.25	36.88	0.806
General health	35.5	15.3	34.1	15.7	−1.4	13.9	−5	22.5	39.8	17.3	42.4	19.4	2.6	17.1	3.75	19.69	0.633

SF36: 36-Item Short Form Health Survey, SD: standard deviation.

*Mann–Whitney U test used for non-normally distributed data.

^a^Clinically meaningful improvement overtime within group (>10% change).

^b^Statistically significant improvement between groups (*p* < 0.05).

#### Lower body strength (5TSTS)

There was no statistically significant difference between the exercise and control group for lower body strength (*p* = 0.574). There were no clinically meaningful improvements over time within each group (Table 2).

#### Lower body endurance (30sSTS)

There was no statistically significant difference between the exercise and control group for lower body endurance (*p* = 0.937). There were no clinically meaningful improvements over time within each group (Table 2).

#### Upper body endurance (30sAC)

There was no statistically significant difference between the exercise and control group for upper body endurance (*p* = 0.121). There were clinically meaningful improvements over time within the exercise (+23%) and control group (+10.7%) (Table 2).

#### Aerobic capacity (2MST)

There was no statistically significant difference between the exercise and control group for aerobic capacity (*p* = 0.745). There were no clinically meaningful improvements over time within each group (Table 2).

### Participant feedback

Participants either strongly agreed or agreed that Zoom was easy to learn and use after the first few sessions. Participants strongly disagreed that they needed someone at home to help them use the system, strongly agreed they were able to use the system by themselves, and rated the audio and video quality as acceptable either all the time or most of the time. Feasibility of telehealth-supervised exercise was high; participants either strongly agreed or agreed they would use it again, were satisfied with the experience, felt safe, and would recommend telehealth to others with SLE. Over half of the participants strongly disagreed or disagreed that they would have preferred to do the exercise sessions on their own without telehealth supervision, and there were mixed responses about whether they would have preferred to go to a central venue instead. Participants strongly agreed or agreed that the exercise program had enough variety, was challenging enough to improve their strength, and that they had sufficient space to perform the exercises at home. The preferred dose parameters were 2 sessions/week (*n* = 5/7, 71%), 30–45 min (*n* = 4/7, 57%) per exercise session, and 8–12 weeks in duration (*n* = 4/7, 57%) (Table 4).

**Table 4.** Quantitative feedback following the telehealth-supervised exercise program (*n* = 7).

Question	Response	*n*, Percentage
Quality of telehealth delivery		
The video quality was acceptable	All of the time	4/7, 57%
	Most of the time	3/7, 43%
The audio quality was acceptable	All of the time	3/7, 43%
	Most of the time	4/7, 57%
Usability of telehealth delivery		
Zoom was easy to learn	Strongly agree	4/7, (57%)
	Agree	3/7, (43%)
After the first few sessions, Zoom was easy to use	Strongly agree	4/7, (57%)
	Agree	3/7, (43%)
I was able to use Zoom on my own.	Strongly agree	7/7, (100%)
I needed someone at home to help me use Zoom.	Strongly disagree	7/7, (100%)
Exercise program satisfaction		
I found the exercises difficult to perform due to my physical ability	Strongly disagree	4/7, (57%)
	Disagree	2/7, (29%)
	Agree	1/7, (14%)
The exercise program was challenging enough to improve my strength	Strongly agree	4/7, (57%)
	Agree	2/7, (29%)
	Agree nor disagree	1/7, (14%)
The exercise program had enough variety	Strongly agree	4/7, (57%)
	Agree	3/7, (43%)
I felt safe doing the exercises	Strongly agree	6/7, (86%)
	Agree	1/7, (14%)
I had enough equipment at home to do the exercises	Strongly agree	7/7, (100%)
I had enough space at home to perform the prescribed exercise program and see the instructor at the same time	Strong agree	6/7, (86%)
	Agree	1/7, (14%)
I would have preferred to do the exercises by myself without being supervised by telehealth	Strongly disagree	2/7, (29%)
	Disagree	3/7, (43%)
	Neither agree nor disagree	2/7, (29%)
Exercise preferences (frequency, time, and duration)		
Preferred length of time for a telehealth-supervised exercise session	30–45 min	4/7, (57%)
	45+ min	3/7, (43%)
Preferred number of telehealth-supervised sessions per week	1 session/week	1/7, (14%)
	2 session/week	5/7, (71%)
	3 sessions/week	1/7, (14%)
Preferred length of time for a telehealth-supervised exercise program	6–8 weeks	1/7, (14%)
	8–12 weeks	4/7, (57%)
	12+ weeks	2/7, (29%)
Satisfaction with telehealth delivery of exercise		
If I had transport, I would have preferred going to a central venue for the sessions (e.g. clinic, community centre, and gym) instead of doing them at home via telehealth	Agree nor disagree	4/7, (57%)
	Strongly agree	1/7, (14%)
	Agreed	1/7, (14%)
	Strongly disagree	1/7, (14%)
Overall, I was satisfied with the telehealth experience	Strongly agree	6/7, (86%)
	Agree	1/7, (14%)
I would recommend telehealth exercise to others who have SLE	Strongly agree	6/7, (86%)
	Agree	1/7, (14%)
I would use telehealth exercise sessions again	Strongly agree	5/7, (71%)
	Agree	2/7, (29%)

Additional information: Note that only the responses that were selected by participants are included in this table for brevity.

### Attendance

Attendance to the exercise program was high (110/112, 98%), with two sessions missed: one due to general malaise, and the other due to a suspected UTI.

## Qualitative results

### Interviews

Four common themes emerged (Table 5).

**Table 5.** Qualitative feedback following the telehealth-supervised exercise program (*n* = 7).

Theme: Ease and efficiency of exercise at home	
*Participant*	*Quote*
8A	‘Not having to commute or travel anywhere, that can take a lot of energy out of me personally, and I’m sure other people with lupus as well. I think that’s a massive positive for telehealth exercise intervention’
17D	‘When you’ve got something like lupus in particular and if you’re immunosuppressed, because I’m on steroids and other people are on those chemo tablets, you don’t really want to be getting public transport into gyms and mingling with lots of people’
17D	‘I had quite a few times where I was really ill and if it was in a different venue other than home, it would have not been happening’
10F	‘Usually, I have to cancel a lot, or I’m too tired, but because I had more energy, I was more alert or felt more awake and I felt like I was able to have a better time with friends’
8A	‘I found the intervention really flexible. So, I think it was really good they were able to accommodate to my preferences and my needs in terms of my scheduling’
29C	‘I think being able to be at home and not having to travel especially with COVID and that kind of thing, knowing you have to get onto public transport just adds that stressful bit right at the beginning’
3E	‘The convenience of it, I suppose, and particularly in light of COVID and getting out and about while having something like lupus, one tends to want to avoid that exposure as much as possible, so that was a big plus’
Theme: Value of live instruction	
*Participant*	*Quote*
10F	‘I feel a lot more safe and secure in what I’m doing, I’m not doubting if it’s wrong because I’m being watched’
	‘Having the exercises two times a week was really helpful, it kind of carries you through’
8A	‘I found the instructors really good, knew what they were doing and knew how to instruct the exercises and do it in a safe way’
	‘Regular sessions pushed me to attend each week’
12B	‘Knowing somebody was going to ask you made you think about your day and how you could fit more exercise into your day’
12G	‘Having the girls ask me twice a week, what have I been doing, made me motivated to actually do things and not just have lazy days all the time’
17D	‘I enjoyed that the sessions were supervised and therefore you had personal encouragement’
3E	‘I was very impressed with the way in which it was executed. The instructors understanding of my particular situation and how they took that into account’
Theme: Challenges of exercising at home	
*Participant*	*Quote*
8A	‘You’re limited with some of the exercises that you can do purely based on what equipment is available’
	‘More variety in the program would be nice’
	‘I think just to have a bit more exposure to different equipment, also potentially having more weights or resistance applied’
12B	‘If we do meet physically, in a different sort of environment, maybe physical contact will actually make me work harder, probably will be more challenging’
	‘The weight I have which is the water bottle could be improved a bit’
	‘I would prefer the exercises to be even more challenging’
10F	‘One thing that I would really like is if you could do a mixture of online and in person’
	‘Maybe I could push myself harder’
	‘I didn’t have proper weights and so it would be nice to have something to hold that’s comfortable’
29C	‘Probably for somebody like me, making it more challenging as we go along, I would have easily coped with that’
3E	‘The only aspect of this delivery is the social contact, not having other people around, that is why I’d like to go to a gym, I feel I really need that social interaction’
12G	‘It took me a while to find a space that would work’
Theme: Continuation of telehealth-supervised exercise sessions	
*Participant*	*Quote*
8A	‘It’s really easy to do, it’s like you can just quickly get changed and be able to do it from home’
12B	‘The convenience of time and flexibility’
29C	‘I think I would probably prefer it versus going to the gym especially with COVID still being the situation it is’
12G	‘I’d like to do it again. It’s easy for me to do because it’s at home’
10F	‘I would be very likely to continue, I asked the instructor if there was any way I could continue’

Theme 1 *Ease and efficiency of exercising at home*. Participants reported that not having to commute to a central venue to exercise was convenient; noting that they would have been more likely to cancel various sessions due to bouts of fatigue. Participants also commented on the positives of being in a comfortable and familiar environment which correlated to high adherence and participant satisfaction. Furthermore, participants who may have been feeling unwell or fatigued prior to an exercise session were still able to safely proceed with their allocated session due to the convenience of it being supervised online, at home.

Theme 2 *Value of live instruction.* Participants reported feeling safe and confident performing the exercises while being supervised online by knowledgeable practitioners. Participants found the practitioners’ communication was clear and encouraging throughout the program. High levels of enjoyment experienced by participants were strongly influenced by the accountability and motivation provided by the individually supervised sessions.

Theme 3 *Challenges of exercising at home*. Participants reported some challenges to exercising at home: lack of physical space in their home and limitations to exercise variability due to lack of equipment. Participants also commented that their personally owned hand weights that they used for the exercise program were either difficult to hold comfortably, or were inadequate in providing enough resistance, emphasising the limitation of exercise equipment.

Theme 4 *Continuation of telehealth-supervised exercise sessions.* Participants reported that they would continue with this exercise delivery mode due to its ease and efficiency. Participants were satisfied with the convenience and flexibility of being able to exercise from home. There were participants who would prefer supervised telehealth exercise over a face-to-face session.

## Discussion

Our main qualitative and quantitative findings suggest that an individually telehealth-supervised exercise program was suitable to and well-accepted by adults with SLE. However, due to a limited number of participants and the possibility that they were more likely to be motivated to exercise and/or have more stable disease, results could exaggerate the true efficacy of the exercise program itself. Recruiting SLE participants was difficult because COVID-19 was of particular concern in Australia during the time of the study, and people with SLE may have been apprehensive about engaging in an exercise trial during this time. It is unclear why there was a lack of male recruitment; however, this is likely because more women have SLE.^43^ Home-based exercise has gathered popularity among practitioners in the past few years due to the COVID-19 pandemic, where this was the only form of exercise delivery, at times. Rapid advancements in mobile technologies have allowed for improvements in intervention delivery and supervision.^44^ Furthermore, face-to-face exercise interventions have shown positive effects on outcomes such as fatigue and QOL in SLE,^24,27,45^ and so, when face-to-face exercise supervision is not an option, it is important that there are feasible alternatives. A decrease in PA and increase in sedentary behaviour during respective lockdowns in response to the COVID-19 pandemic were seen across several populations,^46^ another potential reason for difficulty in recruitment (i.e. less motivation to engage in exercise). Our study, therefore, highlights the potential beneficial effect of telehealth-supervised exercise on outcomes such as fatigue, QOL, and strength in people with SLE. Fatigue is particularly problematic for people with SLE,^3^ with most participants in our study reporting fatigue as a symptom. FACIT-F^47^ was chosen in addition to the SF36 fatigue/energy domain^21^ because FACIT-F is more sensitive to detecting changes in fatigue for people with chronic disease.^19^ Promisingly, both fatigue questionnaires showed a clinically meaningful improvement over time within the exercise group.

To our knowledge, this is the first study to explore telehealth-supervised exercise in SLE using a live video platform. A similar study using a live video to supervise people who have suffered a stroke found high levels of satisfaction with the delivery mode and a high likelihood of participants partaking in supervised telehealth sessions again,^14^ the same result shown in our study. An important theme that emerged from our qualitative assessment was the value of live instruction, enabling safe guidance of exercise and the opportunity for the patient and practitioner to build a rapport. Gherman et al.^48^ indicated that patients who had a good and regular bond with healthcare workers were better at following advice and contributing to their treatment. Another study reveals a strong correlation between higher levels of PA in adults with rheumatoid arthritis when there is live exercise instruction,^49^ which is consistent with our high adherence rate (98%, 110/112 sessions). Furthermore, Wilcox et al.^49^ also highlight the importance of having an instructor who is knowledgeable in the patients’ disease as this is likely to further encourage exercise participation.

An important theme that emerged in our study was the ease and efficiency of exercising at home, with most participants valuing the convenience of not commuting to a centre-based venue. Galloway et al.^14^ indicated that participants favoured the convenience of telehealth as it decreased the burden of transport, a commonly reported barrier for exercise participation in clinical populations. Another study revealed that people with SLE found exercising at home a more comfortable experience.^50^ In our study, we identified a beneficial effect of the exercise program on emotional well-being – it is unclear whether this result can be attributed to the exercise itself, or perhaps because participants received personalised care, attention, and investment from a practitioner during a pandemic lockdown. Regardless of the mechanism of this effect, we suggest that supervised home exercise delivered by telehealth offers holistic benefits for people with a rare disease.

Limitations of this study include low sample size, limiting the statistical credibility of quantitative and qualitative findings; limited number of validated assessments via telehealth, including the SLE disease activity index (SLEDAI) to measure the change in disease activity; non-randomised methodology; inherent lack of blinding; and short duration of exercise, limiting the potential for physiological adaptions.

In this small, exploratory mixed-methods pilot study, we identified that individually supervised telehealth exercise was acceptable, feasible, and satisfying for adults with SLE during a pandemic lockdown. The intervention demonstrated a trend to improvement in perceived QOL, fatigue, and strength outcomes. Although we used data from a previous study to estimate the sample required, our study is underpowered. The effect sizes obtained are modest, and the results, although encouraging, need to be corroborated in a larger, confirmatory investigation, ideally undertaken without the confounding influence of a pandemic and lockdown so that there may be controlled comparison with face-to-face supervised exercise. We recommend that future telehealth-supervised studies include more SLE participants, longer exercise intervention duration, and adopt a randomised and longitudinal study design to measure long-term outcomes.
